# Impact of amalgam dental filling on radiotherapy of head and neck cancer: In vivo dosimetry and dose calculation using AAA and Acuros algorithms

**DOI:** 10.1002/acm2.70378

**Published:** 2025-12-11

**Authors:** Emese Fodor, Zoltán Varga, Gyöngyi Kelemen, Judit Oláh, Anikó Maráz, Katalin Hideghéty

**Affiliations:** ^1^ Department of Oncotherapy University of Szeged Szeged Hungary; ^2^ ELI‐ALPs Non‐profit LTD Szeged Hungary

**Keywords:** AAA and Acuros, dental fillings, film dosimetry, head and neck tumor, IMRT, in vivo dosimetry

## Abstract

**Introduction:**

Dental restorations using high‐density materials can cause inaccuracies in target and organ‐at‐risk (OAR) delineation and dose calculations during radiotherapy. These materials, such as amalgam, lead to dose scattering, resulting in enhanced mucositis in adjacent tissues. Minimizing the impact of these artifacts is crucial to improve dose calculation accuracy. This study evaluates the effects of amalgam tooth fillings on dose distribution, compares two dose calculation algorithms (AAA—anisotropic analytical algorithm and AXB—Acuros XB), and assesses their impact on mucosal toxicity during head and neck radiotherapy.

**Patients and methods:**

Forty‐nine patients with one to five dental amalgam fillings treated with intensity‐modulated radiotherapy (IMRT) for head and neck cancer between 2016 and 2021 at the Oncotherapy Department of Szeged University were included. Planning CTs with and without metal artifact reduction (MAR) were used to delineate targets and OARs. Treatment plans were optimized using the Eclipse Treatment Planning System with the AAA and AXB algorithms. In vivo dosimetry was performed using Gafchromic EBT3 films embedded in thin Styrofoam slabs during one of the first five treatment sessions. Statistical analyses, including *t*‐tests, ANOVA, paired *t*‐tests, and Kaplan–Meier curves, were conducted to evaluate the influence of clinical and dosimetric factors on dose perturbations and mucositis onset.

**Results:**

Metal artifact reduction (MAR) correction improved contouring accuracy. Dose values calculated with AAA were higher than those with AXB for both mean and maximum dose to OARs and mucosa (Dmean: AAA > 10.57%, Dmax: AAA > 6.8% compared to AXB). Measured doses showed better agreement with AAA‐calculated Dmean values (*p* = 0.341) but were significantly underestimated by AXB (*p* < 0.001). There was no difference in dose perturbation according to tumor localization, gross tumor volume, planning target volume, or the use of a tongue wedge. The number of amalgam‐filled teeth correlated significantly with the earlier onset of mucositis, with each additional filling advancing mucositis appearance by 1.7 days.

**Conclusion:**

High‐density dental materials cause significant dose perturbations in the oral cavity during head and neck radiotherapy. In our clinical IMRT setup, the AAA algorithm demonstrated closer agreement with in vivo film measurements compared to AXB, despite the theoretical and literature‐reported superiority of AXB in heterogeneous conditions. This discrepancy underscores that algorithm performance depends on clinical technique, dosimetric methodology, and dose reporting mode. Additionally, each dental filling was associated with earlier mucositis onset.

## 1 INTRODUCTION

In Hungary, for many years, amalgam was the only dental filling material covered by social security for treating dental caries. Amalgam consists of metals with high atomic numbers, including silver, tin, copper, zinc, and mercury. Consequently, many patients over 40 years old likely have amalgam fillings, which can create imaging artifacts and perturb radiation dose distributions during radiotherapy.^1^, ^2^

Metal implants, such as dental fillings, bridges, and other high‐atomic‐number devices, can produce significant imaging artifacts and dose perturbations.^1^, ^3^, ^4^, ^5^ This is particularly relevant in head and neck radiotherapy, where interactions between photons and high‐electron‐density metals increase scatter effects, potentially leading to overdosing in adjacent tissues, especially in surface structures of the oral cavity.^3^, ^4^ Such dose perturbations contribute to radiation‐induced mucositis, which can develop into focal ulcerative lesions, impair nutrition, and cause significant weight loss and discomfort for patients.^6^, ^7^, ^8^, ^9^, ^10^, ^11^, ^12^ Advanced techniques, such as intensity‐modulated radiotherapy, reduce scatter from high‐density dental materials compared to direct fields due to multidirectional, multisegment irradiation.^13^

Accurate dose verification is crucial in these complex anatomical regions. Self‐developing Gafchromic EBT3 films (Ashland, Wilmington, Delaware, USA) are increasingly integral for in vivo dosimetry due to their high spatial resolution, water‐equivalent response, and straightforward handling, making them particularly suitable for anatomically confined areas such as the oral cavity.^14^, ^15^, ^16^, ^17^, ^18^, ^19^, ^20^, ^21^

In this study, Styrofoam slabs were employed as carriers for the films, ensuring reproducible positioning^22^, ^23^ while protecting them from mechanical stress. Styrofoam has been widely applied in radiotherapy for patient immobilization due to its rigid yet easily shaped structure, stability, and favorable absorption properties. It can be considered approximately air‐equivalent, as confirmed by Scherberich et al., Li et al., and Lin et al., highlighting its minimal dosimetric impact.^22^, ^23^, ^24^ This approach follows previous work demonstrating the suitability of Styrofoam for reproducible in vivo dosimetry in small oral cavity regions.^25^, ^26^, ^27^ Patients with dental amalgam fillings gently bit onto the Styrofoam slabs containing the film during the first treatment fraction. A thin 2‐mm Styrofoam layer was placed over the films to prevent direct mucosal contact, which does not significantly affect dose measurement.

The inclusion of in vivo dosimetry served a dual purpose: to provide patient‐specific validation of calculated dose distributions and to complement the comparison of the AAA and AXB algorithms. While both algorithms differ in handling heterogeneities and high‐density materials, they remain computational models that may underestimate or misrepresent dose perturbations caused by dental amalgam. Correlating calculated doses with actual in vivo measurements strengthens confidence in the clinical applicability of algorithm‐derived dose distributions, particularly in challenging geometries with metallic restorations.^25^, ^26^, ^27^

High‐density artifacts from amalgam fillings can distort tumor and organ contours in CT‐based planning, leading to electron density inaccuracies and dose calculation errors, typically underestimating the actual dose near metallic materials. To address these challenges, commercially available dose calculation algorithms such as AAA and AXB are used in clinical practice. AAA is a model‐based algorithm that approximates photon scatter using precomputed kernels and accounts for tissue heterogeneities, while AXB employs a grid‐based deterministic approach solving the linear Boltzmann transport equation, offering higher accuracy in areas with complex density variations, such as around metallic implants.^28^, ^29^

A key aspect of this study was to investigate the effects of implanted metal materials, particularly dental amalgam, on dose distribution in target volumes, adjacent organs, and the oral mucosa. Traditional dosimetric methods often struggle in small anatomical regions, as they may not accurately account for scatter effects from high‐electron‐density materials. In vivo dosimetry allows real‐time, patient‐specific measurements that directly reflect the dose delivered to the oral mucosa and surrounding tissues, providing superior resolution compared to conventional ionization chambers. Furthermore, in vivo measurements address limitations of phantom‐based dosimetry, which often fails to represent patient‐specific anatomical variability. By integrating in vivo dosimetry into the study, we ensured that the planned treatment was accurately delivered, providing a reliable and effective approach to radiotherapy for head and neck cancer patients.

## 2 PATIENTS AND METHODS

### 2.1 Patients

We enrolled 49 patients with one to five dental amalgam fillings who underwent IMRT for head and neck cancer between 2016 and 2021 at University of Szeged (ethical permission no: 51/2024‐SZTE IKEB). Patients were positioned supine using the AIO system (ORFIT Industries) with a head holder and a four‐point thermoplastic mask fixation. A Styrofoam tongue wedge was applied in 47 cases.

### 2.2 Imaging and contouring

CT imaging covered the entire head and neck from the skull apex to the T3–T4 vertebrae, with slice thicknesses of 2.5–5 mm. Metal Artifact Reduction algorithms were applied to minimize distortions caused by dental fillings. PET‐CT and MRI scans were co‐registered and fused with the planning CT to delineate the gross tumor volume (GTV) accurately. Planning target volumes (PTVs) and organs at risk (OARs) were contoured following NCCN Guidelines (1.2015, 1.2016, 2.2020). Amalgam fillings were contoured, and their density was set to 2.7–3.1 g/cm^3^ for dose calculation purposes. Selected OARs for dose comparison included the mandible, buccal mucosa, oral cavity, upper constrictor muscle, and lips, based on ESTRO (2015) and NCCN (2016) recommendations. Additional dose calculations were performed for the film and a 1 cm^2^ mucosal area adjacent to the dental fillings.

### 2.3 Treatment planning

Standard five‐field IMRT plans were generated with gantry angles of 0° (collimator 5°, table 0°), 72° (collimator 5°, table 0°), 144° (collimator 90°, table 0°), 216° (collimator 355°, table 0°), and 288° (collimator 90°, table 0°). Dose calculations were performed using the Eclipse Treatment Planning System (ver. 18, Varian Medical Systems, Palo Alto, California, USA) with two algorithms: AAA and AXB, employing a 1.5 mm calculation grid and 1.25 mm optimization resolution. IMRT used a dynamic sliding window technique. Final plans were normalized to ensure that 95% of the PTV received the prescribed dose (50.4 Gy/28 fx), and the “normal tissue objective” function (priority 110) was used to minimize OAR exposure. Dose coverage to PTV was assessed via mean, modal, minimum, and maximum doses, and DVHs were evaluated for both algorithms. The percentage difference between AAA and AXB was calculated as X = (AAA−AXB)/AXB. Final plan approval was based on comprehensive evaluation of quantitative metrics and DVH comparisons for all 49 patients.

### 2.4 Quality assurance

All plans were verified before treatment using phantom measurements and portal dosimetry. Treatments were delivered on consecutive days, with ConeBeam CT (CBCT) used prior to each fraction for setup verification.

### 2.5 In vivo dosimetry

**FIGURE 1:**
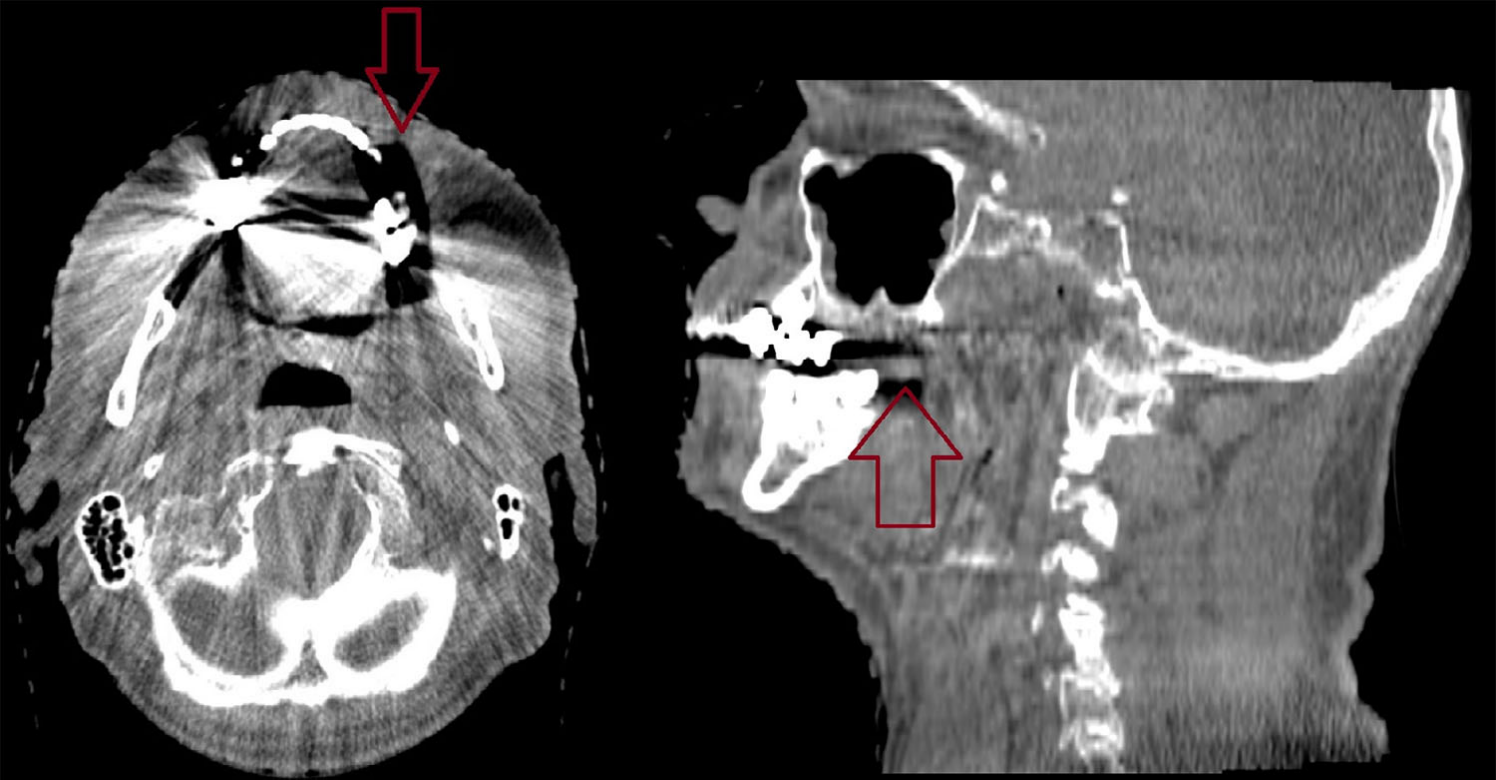
The locations of the amalgam dental fillings and the films (red arrow) on axial and sagittal CT slice.

**FIGURE 2:**
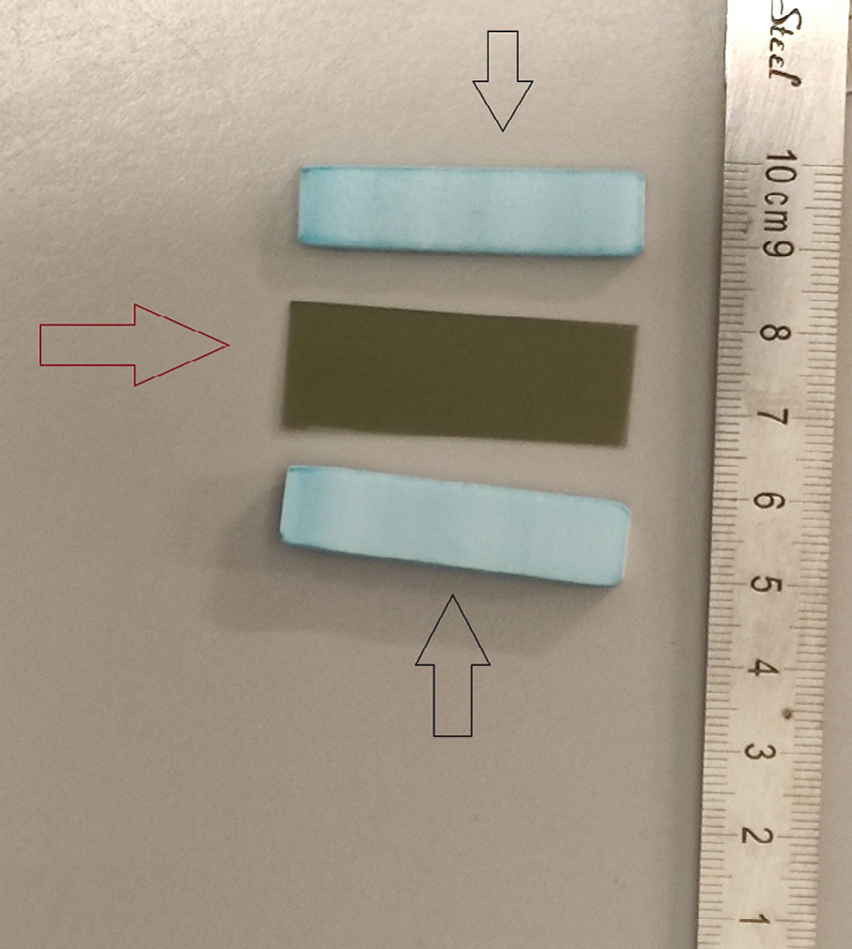
Films (red arrow) were covered with Styrofoam (black arrow) of 2 mm in thickness.

After informed consent, a Gafchromic EBT3 film (1 × 5 cm) was positioned intraorally between two thin Styrofoam layers, and the patient gently bit on the device over the target amalgam restoration during the first treatment fraction (Figure 1). Regardless of the number of amalgam teeth in the patients, we only placed a film on one amalgam tooth in each patient. The film was covered with a 2 mm Styrofoam layer to prevent mechanical stress and direct mucosal contact (Figure 2).

A CBCT scan was acquired with the film in place and registered to the planning CT dataset. The film location was contoured on the axial plane, verified in the sagittal view, and defined as a finite 3D ROI, considering its physical thickness (∼275 µm) and surface area. Dose distributions were recalculated with both AAA and AXB algorithms and mean and distribution of doses within the ROI were compared with the measured values obtained from ImageJ analysis of the irradiated films. To account for CBCT exposure, a 2% adjustment was applied to all measured values.

Film calibration was performed using a Varian TrueBeam linear accelerator in a 30 × 30 cm solid water phantom (SSD 100 cm, depth 1.5 cm, 6 MV photons, 300 MU/min, 10 × 10 cm^2^ field) with calibration doses ranging from 0 to 2.5 Gy. Films were scanned using an Epson Expression 10000XL flatbed scanner (TIFF, 72 dpi).

### 2.6 Statistical analysis

Statistical analyses were performed using SPSS version 26.0. Time to onset of mucositis relative to the number of filled teeth was analyzed using the Kaplan–Meier method. Differences between AAA and AXB plans were assessed using *t*‐tests and ANOVA, with *p* < 0.05 considered statistically significant. Regression analysis evaluated the relationship between the number of filled teeth and mucositis onset.

## 3 RESULTS

The patient characteristics are summarized in Table 1. The most common tumor sites were the mesopharynx (51%, *n* = 25), the base of the hypopharynx (24.5%, *n* = 12), the larynx (20.4%, *n* = 10), and the parotid (4.1%, *n* = 2). The patients' ages ranged from 35 to 85 years, with a median age of 62.2 years. The distribution of dental fillings was as follows: second canine (*n* = 28), middle incisors (*n* = 8), and second premolar (*n* = 13).

**TABLE 1:** Patient‐ and tumor‐related characteristics of the study population.

Characteristics		Number of patients
		*n* = 49 (%)
Median age (range), years		62.2 (35–85)
Gender (%)	Male	42 (85.7)
	Female	7 (14.3)
Tumor sites	Mesopharynx	25 (51.0)
	Hypopharynx	12 (24.5)
	Larynx	10 (20.4)
	Parotid	2 (4.1)
Distribution of dental fillings (%)	Second canine	28 (57.2)
	Middle incisors	8 (16.3)
	Second premolar	13 (26.5)

### 3.1 Analysis representing the place of the film

Mean and maximum values were significantly different between AAA and AXB. The average dose is 10.57%, while the maximum dose is 6.48% higher on average.

### 3.2 In vivo dosimetry

Measured doses showed good agreement with AAA‐calculated Dmean (*p* = 0.341), but were significantly higher than AXB‐calculated Dmean (*p* < 0.001) (Table 2).

**TABLE 2:** Mean and maximum dose for OAR's calculated by the AAA and AXB algorithms and the measured and calculated mean doses on the amalgam tooth.

OAR	Mean dose (mean ± SD, Gy)				Maximum dose (mean ± SD, Gy)		
	AAA	AXB	Image J	p	AAA	AXB	p
Lips	20.82 ± 7.00	20.58 ± 7.20	NA	0.024	36.75 ± 8.71	36.47 ± 8.89	0.184
Mandible	35.31 ± 6.75	34.21 ± 6.77		<0.001	53.14 ± 1.52	52.53 ± 1.93	<0.000
Upper musculus constrictor	48.94 ± 5.45	48.63 ± 5.45		<0.001	52.85 ± 0.51	52.81 ± 0.78	0.653
Buccal mucosa left	33.34 ± 8.60	32.75 ± 8.61		0.001	47.56 ± 7.16	47.11 ± 7.30	0.012
Buccal mucosa right	33.44 ± 9.29	32.92 ± 9.48		0.001	47.05 ± 7.73	46.44 ± 8.00	0.013
Oral cavity	38.06 ± 5.56	37.67 ± 5.43		<0.001	52.30 ± 1.65	52.03 ± 1.61	0.008
On amalgam tooth	1.24 ± 0.33	1.13 ± 0.33	1.21 ± 0.31	<0.001	1.70 ± 0.26	1.61 ± 0.25	<0.001

Effect of target volumes on the calculated dose of the applied gafchromic film smaller PTVs (< 670 cm^3^) resulted in a greater difference in film Dmean (13.23% vs. 8.02%, *p* = 0.090), although this did not reach significance. However, smaller GTVs (< 50 cm^3^) showed a significant difference in film Dmax (9.91% vs. 3.19%, *p* = 0.022). The above percentage increases were significant regarding different tumor locations. They did not differ in the mean or maximum dose of PTV in the tumour location, but the pairwise comparison showed that the hypopharynx is the only exception (16.88%, *p* = 0.066).

### 3.3 Styrofoam wedge

A custom‐made Styrofoam wedge was used in 37 cases to increase the distance between the tongue and hard palate. No significant difference in doses calculated by AAA and AXB was observed with the wedge compared to without the wedge (at PTV max dose: 7.58% vs. 6.1%, *p* = 0.674 and at PTV mean dose: 14.2% vs. 9.4%, *p* = 0.362).

### 3.4 Comparative indexes

The mean and maximum doses (Dmean and Dmax) calculated by the AAA and AXB algorithms were compared for several organs at risk (OARs), including the mandible, buccal mucosa, oral cavity, upper musculus constrictor, and lips. Dmean and Dmax for OARs were significantly higher with AAA compared to AXB, except for Dmax of the lips (*p* = 0.184) and Dmax of the upper pharyngeal constrictor (*p* = 0.653) (Table 2).

### 3.5 Effect of target volumes on OAR dose

The size of the target volumes had no effect on the dose differences of the OARs. Tumor localization had no impact on the OAR dose calculation differences.

### 3.6 Side effect onset

**FIGURE 3:**
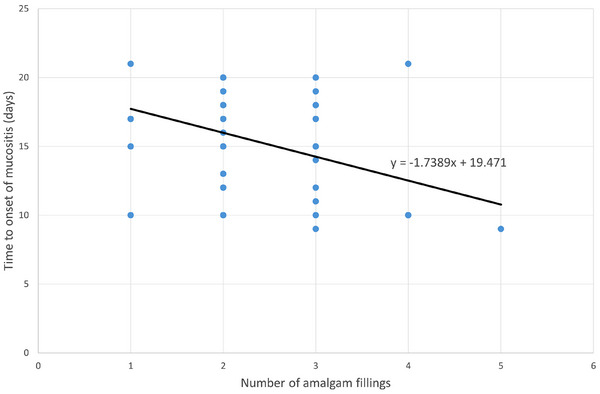
The number of amalgam fillings correlated with the onset of mucositis.

The number of amalgam fillings correlated with the onset of mucositis. Patients with more filled teeth experienced mucositis earlier. Regression analysis showed that each additional filled tooth led to mucositis onset 1.7 days earlier (Figure 3).

Grade I and grade II radiodermatitis was observed in 25 and 24 cases, respectively. Radiomucositis developed in five patients. Irradiation had to be ceased in 34 cases. Mean of the therapeutic break was 8.6 days. Due to the low number of cases, we could not find any association between the presence or severity of side effects, the necessity of a therapeutic break and patients' characteristics (number and place of dental filling, use of wedge).

## 4 DISCUSSION

Although many studies have compared AAA and AXB for tumor sites such as lung,^30^, ^31^, ^32^, ^33^, ^34^ nasopharyngeal carcinoma,^35^, ^36^, ^37^ and breast cancer,^38^, ^39^ no intercomparison has specifically addressed the impact of metal implants in water‐equivalent surroundings, such as the oral cavity.

The results of our study are consistent with existing literature regarding the effects of dental amalgam fillings on radiation dose distribution during IMRT for head and neck cancer. Previous studies have demonstrated that metal objects, such as dental fillings, can create artifacts in the dose distribution, affecting the accuracy of radiation delivery to adjacent tissues. Our findings, which show that the presence of dental fillings significantly alters the dose to critical OARs, are in line with other studies such as those by Pegah Saadatmand et al.,^40^ who observed similar dose discrepancies in the presence of metal. The position of dental fillings plays a significant role in modifying radiation dose distribution, as demonstrated in our study. The lips and musculus constrictor, located farther from the dental fillings, experienced less scatter and inhomogeneity in dose distribution. This observation aligns with findings from Chin et al.,^41^ who noted that regions farther from dental metals are less influenced by dose artifacts. Additionally, the size of the tumor, specifically a larger GTV (> 50 cm^3^), enhances the interaction between photons and the high electron density of the metal, resulting in dose distortions, as confirmed by studies like those from Yusung et al.^42^ Furthermore, the difference in OAR Dmean was more pronounced for smaller PTVs (≤670 cm^3^), especially when OARs were near bone or air. These results support the work of Drosoula Giantsoudi et al.,^43^ who observed that smaller treatment volumes can amplify the impact of metal artifacts, particularly when adjacent to high‐density structures like bone. Together, these findings highlight the importance of considering both the position of dental fillings and tumor volume in treatment planning to minimize dose inaccuracies.^44^, ^45^, ^46^

In our study, we compared the results from the AAA and AXB algorithms for dose calculation, and found that the AAA algorithm consistently overestimated the dose to the OARs, particularly in regions near the dental fillings. This result mirrors findings in the literature where AAA has been shown to be less accurate in heterogeneous media, especially in the presence of high electron density materials like dental amalgam.^28^ On the other hand, AXB, which models dose deposition by solving the linear Boltzmann transport equation, performed more accurately in the regions around metal, as seen in our analysis of OARs such as the mandible, buccal mucosa, and oral cavity. This supports the conclusions of Tao Han et al.,^47^ who also noted that AXB offers superior accuracy in heterogeneous environments compared to other algorithms.

One of the more interesting observations from our study is the relationship between the number of dental fillings and the onset of mucositis, a common side effect in patients undergoing radiotherapy for head and neck cancer. Our regression analysis showed that for each additional dental filling, the appearance of mucositis occurred 1.7 days earlier. This finding corroborates the results of study by Hiroaki Shimamoto et al.^48^ who similarly reported that patients with more dental fillings experienced a higher incidence and earlier onset of radiation‐induced mucositis. This can be attributed to the scatter effects from the metal, which may lead to higher local doses to mucosal tissues near the fillings.

Our study also found that the use of Styrofoam wedges to increase the distance between the oral cavity structures did not affect the dose distribution significantly, as evidenced by the comparable OAR doses in patients with and without the wedge. This finding contrasts with studies by Herpel et al.,^49^ who suggested that individual tissue retractor could potentially reduce dose to the oral cavity. However, this discrepancy might be due to differences of using a simple custom Styrofoam wedge, against an individually designed 3D printed tissue retractor. In addition, this result confirms that the styrofoam composition is favorable, as its water‐equivalent thickness of 2 mm does not significantly increase the surface dose.^24^

In external radiotherapy, in vivo dosimetry has become a widely accepted quality assurance method to compare planned versus delivered doses during treatment.^50^, ^51^ Ionization chambers, while effective in larger fields, may not provide reliable measurements for very small fields due to their physical size.^52^ In contrast, self‐developing films offer superior resolution and gamma analysis capabilities, making them ideal for small‐field dosimetry, such as in stereotactic radiotherapy.^53^ Films are quasi energy‐independent over MV photon energies, which is beneficial when high doses are delivered.^52^ Given their precise performance and cost‐effectiveness, films should be considered in cases where ionization chambers are impractical. They do not require calibration at regular intervals, unlike ionization chambers.^53^ In terms of in vivo dosimetry in our study, use of patient films placed directly over the dental fillings provides real‐world data that is more accurate than phantom‐based dosimetry, which often involves assumptions that may not account for patient‐specific variables. This approach has been endorsed by several authors, such as Farhat et al.^50^ and Fan‐Chi Su,^14^ who emphasized the importance of in vivo measurements for quality assurance in clinical practice.

In line with previous reports, AXB theoretically provides superior accuracy in heterogeneous media, particularly around high‐density materials, due to its deterministic solution of the Boltzmann transport equation.^54^, ^55^ However, in our clinical IMRT setup with fixed fields, the measured EBT3 film doses showed closer agreement with AAA than AXB. This finding does not contradict the established superiority of AXB but rather highlights that dose agreement is influenced by multiple factors, including the irradiation technique (IMRT vs. VMAT), the definition of dose reporting (dose‐to‐water vs. dose‐to‐medium).^56^, ^57^, ^58^ Since EBT3 films are calibrated in dose‐to‐water, their readings may align more closely with AAA, which reports dose‐to‐water by default, whereas AXB provides higher physical accuracy in dose‐to‐medium. Consequently, while AXB remains the more robust algorithm in principle, our results demonstrate that in specific IMRT setups AAA may coincidentally align more closely with in vivo film measurements. These observations emphasize the importance of considering both the calculation algorithm and the delivery technique when evaluating the dosimetric impact of dental amalgam fillings.

## 5 CONCLUSION

This study highlights the significant influence of dental amalgam fillings on radiation dose distribution in head and neck cancer treatments. Our findings confirm that metal fillings, particularly in proximity to critical structures, can distort the dose distribution, with the effect becoming more pronounced in smaller target volumes or regions involving bone or air. Among the algorithms tested, AXB generally demonstrated superior accuracy by effectively accounting for the scattering effects caused by high‐density materials, offering an advantage over conventional algorithms.

The use of in vivo dosimetry in this study proved invaluable, particularly in the unique context of the oral cavity, where the presence of small amalgam fillings presents a challenge for traditional dosimetric tools. By directly measuring the delivered dose in real patients, we were able to validate the accuracy of treatment planning and account for individual anatomical variations.

Our results reinforce the principle that no single algorithm is universally superior; rather, their performance must be evaluated in the context of specific clinical techniques, dosimetric methods, and patient anatomies. These findings underscore the importance of advanced dosimetric techniques and sophisticated algorithms in ensuring precise and effective radiation therapy, particularly in complex cases with implanted metal materials.

## AUTHOR CONTRIBUTIONS

**Emese Fodor**: Conceptualization; Measurements; Data Curation; Visualization; Writing Original draft; **Zoltán Varga**: Measurements; Writing Review & Editing; **Gyöngyi Kelemen**: Contouring; Writing Review & Editing; **Judit Oláh**: Supervision; **Anikó Maráz**: Conceptualization; Writing Review & Editing; **Katalin Hideghéty**: Methodology; Writing Review & Editing.

## CONFLICT OF INTEREST STATEMENT

The authors have no relevant conflicts of interest to disclose.
